# Dextran-Coated Iron Oxide Nanoparticles Loaded with Curcumin for Antimicrobial Therapies

**DOI:** 10.3390/pharmaceutics14051057

**Published:** 2022-05-14

**Authors:** Cristina Chircov, Raluca-Elena Ștefan, Georgiana Dolete, Adriana Andrei, Alina Maria Holban, Ovidiu-Cristian Oprea, Bogdan Stefan Vasile, Ionela Andreea Neacșu, Bianca Tihăuan

**Affiliations:** 1 Department of Science and Engineering of Oxide Materials and Nanomaterials, University Politehnica of Bucharest, 011061 Bucharest, Romania; cristina.chircov@yahoo.com (C.C.); dolete.georgiana@gmail.com (G.D.); bogdan.vasile@upb.ro (B.S.V.); 2 National Research Center for Micro and Nanomaterials, University Politehnica of Bucharest, 060042 Bucharest, Romania; ovidiu.oprea@upb.ro; 3 Faculty of Engineering in Foreign Languages, University Politehnica of Bucharest, 011061 Bucharest, Romania; ralucastefan98@yahoo.com; 4 Microbiology and Immunology Department, Faculty of Biology, Research Institute of the University of Bucharest, University of Bucharest, 060101 Bucharest, Romania; andrei.adriana@s.bio.unibuc.ro (A.A.); alina_m_h@yahoo.com (A.M.H.); 5 Department of Inorganic Chemistry, Physical Chemistry and Electrochemistry, University Politehnica of Bucharest, 1–7 Polizu Str., 011061 Bucharest, Romania; 6 National Research Center for Food Safety, University Politehnica of Bucharest, 060042 Bucharest, Romania; 7 Research Institute of the University of Bucharest—ICUB, 91–95 Spl. Independentei, 50567 Bucharest, Romania; bianca.tihauan@sanimed.ro; 8 Research & Development for Advanced Biotechnologies and Medical Devices, SC Sanimed International Impex SRL, 087040 Călugăreni, Romania

**Keywords:** iron oxide nanoparticles, microwave-assisted hydrothermal method, dextran, curcumin, antimicrobial therapy

## Abstract

The current trend in antimicrobial-agent development focuses on the use of natural compounds that limit the toxicity of conventional drugs and provide a potential solution to the antimicrobial resistance crisis. Curcumin represents a natural bioactive compound with well-known antimicrobial, anticancer, and antioxidant properties. However, its hydrophobicity considerably limits the possibility of body administration. Therefore, dextran-coated iron oxide nanoparticles can be used as efficient drug-delivery supports that could overcome this limitation. The iron oxide nanoparticles were synthesized through the microwave-assisted hydrothermal method by varying the treatment parameters (pressure and reaction time). The nanoparticles were subsequently coated with dextran and used for the loading of curcumin (in various concentrations). The drug-delivery systems were characterized through X-ray diffraction (XRD) coupled with Rietveld refinement, transmission electron microscopy (TEM), high-resolution TEM (HR-TEM), selected area electron diffraction (SAED), dynamic light scattering (DLS) and zeta potential, thermogravimetry and differential scanning calorimetry (TG-DSC), vibrating sample magnetometry (VSM), and UV-Vis spectrophotometry, as well as regarding their antimicrobial efficiency and biocompatibility using the appropriate assays. The results demonstrate a promising antimicrobial efficiency, as well as an increased possibility of controlling the properties of the resulted nanosystems. Thus, the present study represents an important step forward toward the development of highly efficient antimicrobial drug-delivery systems.

## 1. Introduction

Microbial pathogen infections represent a continuously growing problem with severe worldwide implications, as they affect millions of lives daily and represent a major death cause in both adults and children [1,2]. Although antibiotics significantly reduced mortality rates associated with infections, their overuse has resulted in the emergence of the antimicrobial resistance crisis [3,4,5]. Current strategies focus on the use of alternative antimicrobial agents and substances to counteract the negative effects of antimicrobial resistance.

There has been an increasing amount of scientific interest in the application of natural bioactive substances that exhibit antimicrobial properties. Medicinal plant extracts can inhibit the growth of bacteria, viruses, fungi, and protozoa, with a significant clinical value in the fight against antibiotic-resistant species [6,7,8,9]. Curcumin, also known as diferuloylmethane, is the flavonoid compound of turmeric with confirmed antimicrobial, anticarcinogenic, antioxidant, and anti-inflammatory properties [10,11,12,13,14,15,16]. Pharmacologically, curcumin is a lipophilic diketone that is poorly absorbed [17,18]. Therefore, its direct administration inside the organism is challenging and consequently requires suitable pharmaceutic formulations.

In this context, iron oxide nanoparticles represent a promising option for the development of drug-delivery systems for the controlled release of natural substances. Besides providing suitable nanostructured support for the efficient loading of curcumin, iron oxide nanoparticles have often demonstrated intrinsic antimicrobial properties [19,20,21,22]. In this manner, by combining iron oxide nanoparticles and curcumin, a synergistic antimicrobial therapy could be achieved [23]. While iron oxide nanoparticles are usually synthesized through the coprecipitation method, the properties of the so-obtained nanoparticles are poor in terms of crystallinity and stability. Moreover, the coprecipitation method does not allow the control of nanoparticle size and size distribution [24]. Thus, the microwave-assisted hydrothermal method has emerged as a promising technique that could potentially overcome the associated issues [25].

In order to increase the biocompatibility and stability of the iron oxide nanocarriers, as well as the curcumin loading capacity, an organic coating can be applied. Dextran is a natural biodegradable exopolysaccharide consisting of glucose subunits that is biosynthesized by the nonpathogenic *Leuconostoc mesenteroides* bacterium [26,27]. Dextran is generally known for its anti-inflammatory and antithrombotic properties, and the functional hydroxyl groups within its structure provide a facile means for conjugations with other substances [28].

In this study, novel iron oxide nanocarriers were developed by varying the synthesis parameters (i.e., pressure, reaction time) of the microwave-assisted hydrothermal method, followed by their coating with a dextran layer, in order to stabilize and increase the efficiency of curcumin loading, added in different concentrations.

## 2. Materials and Methods

### 2.1. Materials

Ferrous sulfate heptahydrate (FeSO_4_·7H_2_O), ferric chloride hexahydrate (FeCl_3_·6H_2_O), ammonium hydroxide 25% (NH_4_OH), ethanol, and curcumin were purchased from Sigma-Aldrich Merck (Darmstadt, Germany). Dextran (M~500,000 g/mol) and phosphate buffer saline (PBS) were purchased from Carl Roth (Karlsruhe, Baden-Württemberg, Germany). All chemicals were of analytical purity and used with no further purification.

The antimicrobial assays were performed using three microbial strains (*Escherichia coli* ATCC 25922, *Staphylococcus aureus* ATCC 25923, and *Candida albicans* ATCC 10231) obtained from the collection of the microbiology laboratory within the Faculty of Biology, University of Bucharest.

HT-29 human epithelial cells (colon adenocarcinoma, well-differentiated) (ATCC, Merck, Romania) were selected as the model for cytotoxicity assessments.

### 2.2. Methods

#### 2.2.1. Synthesis of Dextran-Coated Iron Oxide Nanoparticles Loaded with Curcumin

The synthesis of the drug-delivery systems involved three distinct steps. Initially, the iron oxide nanoparticles were obtained through the microwave-assisted hydrothermal method, similar to one of our previous studies [29]. Briefly, FeSO_4_·7H_2_O and FeCl_3_·6H_2_O were dissolved in ultrapure water (1:2 molar ratio), and the solution was dripped into an alkaline solution of NH_4_OH using a peristaltic pump. The obtained black precipitate was transferred into a polytetrafluoroethylene (Teflon) vial and further introduced into the Milestone Synthwave equipment for microwave-assisted hydrothermal treatments. Four different parameter regimes were applied (as depicted in Table 1), thus resulting in four types of iron oxide nanoparticle supports. The nanoparticles were allowed to naturally cool to room temperature, decanted with a NdFeB magnet, and washed with ultrapure water until a neutral pH.

Subsequently, the iron oxide nanoparticles were redispersed in ultrapure water, followed by the addition of 10 wt% dextran. The obtained mixture was kept under continuous magnetic stirring for 12 h at room temperature. The coated nanoparticles were magnetically separated and dried overnight at 40 °C. 

The final stage consisted of the milling of the nanoparticles in a ceramic mortar using ethanol to prevent oxidation and their redispersion in 50 mL ethanol, where 1, 5, and 10 wt% of curcumin was dissolved. The mixtures were magnetically stirred for 12 h at room temperature. The nanoparticles were collected via centrifugation at 6000 rpm for 10 min and dried overnight at 40 °C, while the supernatant was kept for further analyses.

#### 2.2.2. Physicochemical Characterization of Dextran-Coated Iron Oxide Nanoparticles Loaded with Curcumin

##### X-ray Diffraction (XRD) Coupled with Rietveld Refinement

A PANalytical Empyrean diffractometer (PANalytical, Almelo, The Netherlands) equipped with CuK_α_ radiation of λ = 1.541874 Å, a hybrid monochromator 2 × Ge (220) for Cu, and a parallel-plate collimator on the PIXcel3D detector was used for the XRD analysis. Scanning was performed within the 2 θ angle range between 10 and 80°, with an incidence angle of 0.5°, step size of 0.0256°, and time for each step of 1 s. The Rietveld refinement was performed using the HighScore Plus software (version 3.0, PANalytical, Almelo, The Netherlands) for assessing the nanoparticle crystallinity and crystallite size.

##### Transmission Electron Microscopy (TEM). High-Resolution TEM (HR-TEM). Selected Area Electron Diffraction (SAED)

A small sample amount was dispersed into deionized water, and 10 µL of the suspension was placed on a 400-mesh lacey carbon-coated copper grid at room temperature. The samples were analyzed using a high-resolution 80–200 TITAN THEMIS transmission microscope (purchased from FEI, Hillsboro, OR, USA) equipped with an Image Corrector and EDXS detector in the column. The microscope is operated at a 200 kV voltage in transmission mode. Particle-size distribution was assessed by creating histograms corresponding to the TEM images using the ImageJ software (University of Wisconsin, Madison, WI, USA). 

##### Fourier Transform Infrared (FT-IR) Spectroscopy

A Thermo iN10-MX Fourier transform (FT)-IR microscope (Thermo Fischer Scientific, Waltham, MA, USA) with a liquid nitrogen-cooled mercury cadmium telluride detector was used for obtaining the IR spectra. The measurement was performed in reflection mode in the range of 4000–400 cm^−1^ and at a resolution of 4 cm^−1^. 64 scans were co-added and converted to absorbance using the OmnicPicta software (Thermo Scientific) for each sample.

##### Dynamic Light Scattering (DLS) Zeta Potential

For determining the hydrodynamic diameter and the surface charge of the nanostructured systems, the samples were dispersed in deionized water (~6.9 pH) at a concentration of 0.3 mg/mL. For each sample, five acquisitions were measured using the DLS technique (DelsaMax Pro, Backman Coulter, Brea, CA, USA).

##### Thermogravimetry and Differential Scanning Calorimetry (TG-DSC)

The thermogravimetric analysis was performed using the STA TG/DSC Netzsch Jupiter 449 F3 equipment (Selb, Germany). The samples were placed in an alumina crucible and subjected to the thermal treatment with a temperature range between 20 and 900 °C (heating rate of 10 K/min) in a dynamic air atmosphere of 50 mL/min.

##### Vibrating Sample Magnetometry (VSM)

The magnetic properties of the dextran-coated iron oxide nanoparticles were determined at room temperature (25 °C) through VSM analysis (VSM, VersaLabTM 3T, Cryogen-free Vibrating Sample Magnetometer, Westerville, OH, USA). The magnetic field applied ranged between −10 and +10 kOe two times, with a step rate of 10 Oe/s.

##### UV-Vis Spectrophotometry

The UV-Vis spectrophotometry measurements were performed using a Thermo Evolution 600 double-beam UV-Vis spectrophotometer (Thermo Fischer Scientific, Waltham, MA, USA). Initially, the quantity of the unloaded curcumin within the supernatant was determined by UV-Vis spectrophotometry at a fixed wavelength (λ = 428 nm) using a standard cuvette with an optical path of 1 cm. In this manner, the drug loading efficiency was calculated using the following equation:

(1) $$\mathrm{drug}\;\mathrm{loading}\;\mathrm{efficiency}\;(\%)=\frac{\mathrm{the}\;\mathrm{total}\;\mathrm{amount}\;\mathrm{of}\;\mathrm{drug}\;-\;\mathrm{free}\;\mathrm{amount}\;\mathrm{of}\;\mathrm{drug}}{\mathrm{the}\;\mathrm{total}\;\mathrm{amount}\;\mathrm{of}\;\mathrm{drug}}\;\times \;100$$

Finally, the curcumin release was investigated in a PBS:ethanol = 3:2 (*v*/*v*) solution at a pH of 8.11. For each sample, 100 mg of the nanoparticles were introduced into a filter bag enclosure and placed into 25 mL of the solution, under continuous stirring at 37 °C. The measurements were performed in a continuous flow regime using a Hellma™ Quartz UV flow cell (100 µL volume, 3 mm path length), for assessing the initial short-term release (4 h), followed by measurements at established timepoints for assessing the long-term release (8 h, 12 h, 24 h, 48 h, and 7 days). The obtained results were further used for estimating the release of curcumin using the following equation:

(2) $$\mathrm{curcumin}\;\mathrm{release}\;(\%)=\frac{\mathrm{the}\;\mathrm{amount}\;\mathrm{of}\;\mathrm{released}\;\mathrm{curcumin}}{\mathrm{the}\;\mathrm{total}\;\mathrm{amount}\;\mathrm{of}\;\mathrm{loaded}\;\mathrm{curcumin}\;}\;\times \;100$$

##### Antimicrobial-Activity Assay

The protocols were performed according to previous antimicrobial studies [30,31]. The antimicrobial effect was assessed for the pristine iron oxide controls, dextran-coated, and curcumin-loaded dextran-coated iron oxide nanoparticles.

UV-sterilized nanoparticles and control powders were diluted in sterile distilled water/DMSO to obtain a stock solution of 1 mg/mL concentration. Qualitative antimicrobial evaluation was performed by an adapted diffusion test from the Clinical & Laboratory Standards Institute Guidelines. Briefly, Petri dishes containing Mueller–Hinton medium (or Sabouraud Dextrose Agar for the yeast, *Candida albicans*) were swab-inoculated with a microbial suspension of 1–3 × 10^8^ CFU/mL (CFU—colony-forming units), corresponding to 0.5 MacFarland density standard. Stock solutions of the obtained nanoparticles and controls were added dropwise (10 µL) on the swab-inoculated plates. The Petri dishes were incubated for 24 h at 37 °C. Subsequently, the inhibition zone diameter was measured, with results expressed in mm.

For assessing the minimum inhibitory concentration (MIC), a microdilution technique in 96-well plates was performed. Serial binary dilutions (from 2 mg/mL to 0.0625 mg/mL) were obtained for each of the tested nanoparticles and controls in nutritive broth and a bacterial inocula of ~10^6^ CFU/mL was utilized. The cultures were incubated for 24 h at 37 °C. The minimum inhibitory concentration (MIC) was quantitatively determined by measuring the Optical Density (OD 620 nm) of the obtained cultures with a spectrophotometer and by naked-eye analysis.

The efficiency of biofilm inhibition was assessed by a crystal violet-adapted protocol performed in microvolumes, in 96-well plates. Serial binary dilutions (from 2 mg/mL to 0.0625 mg/mL) were obtained for each of the tested nanoparticles and controls in nutritive broth and a bacterial inocula of ~10^6^ CFU/mL was utilized. The plates were incubated for 24 h at 37 °C, washed with sterile physiological water, and fixated with cold methanol for 5 min. The dried plates were stained with 1% crystal violet solution for 20 min, and after washing the excess with tap water, the stain within the biofilm was solubilized with a 33% solution of acetic acid. The biofilm inhibitory concentration (BIC) was quantitatively determined by measuring the absorbance (at 492 nm) of the obtained cultures with a spectrophotometer.

##### Biocompatibility Assay

The MTT assay was used for measuring the cellular metabolic activity and as an indicator of cell viability, proliferation, and cytotoxicity. All samples were dissolved in DMSO (Dimethyl sulfoxide 99%, Merck, Romania) and analyzed at a 10 mg/mL concentration. Control samples represented by iron oxide nanoparticles without the dextran coating were tested at the same concentrations as the samples. A negative control containing cells treated with DMSO was also included. HT-29 cells were cultivated in RPMI-1640 culture medium (Sigma-Aldrich, St. Louis, MO, USA) and supplemented with 2 mM Glutamine (Sigma-Aldrich), 10% heat-inactivated Fetal Bovine Serum (FBS) (Sigma-Aldrich), and 1% Pen/Strep (penicillin/streptomycin solution, 50 µg/mL, Sigma-Aldrich) for 24 and 48 h at 37 °C, 95% humidity with 5% CO_2_. After the 24 and 48 h time points, the cells were washed with PBS, harvested using trypsin (Sigma-Aldrich), and counted using Trypan Blue (Sigma-Aldrich) and a hemocytometer. The seeding density for the MTT assays was optimized at 5 × 10^5^. 

Cells seeded at 5 × 10^5^ density in a clear 96-well cell-culture plate were treated with the 5% curcumin samples and the controls and incubated for 24 and 48 h at 37 °C, 95% humidity with 5% CO_2_. After 24 and 48 h of exposure, the cells were treated with MTT solvent (Roche, Basel, Switzerland) for 15 min at room temperature. The absorbance was measured using a spectrophotometric microplate reader (ELISA reader) at OD = 570 nm.

##### Statistical Analysis

All biological experiments were performed in triplicate. Data are represented as mean ± standard deviation (S.D.). The statistical analysis was performed using the GraphPad Prism 9 software (San Diego, CA, USA). Data were compared using one-way analysis of variance (ANOVA), followed by a two-tails *t*-test with Bonferroni post hoc correction. The level of significance was set to *p* < 0.05/n.

## 3. Results

The iron oxide nanoparticles, which act as the main supports of the present drug-delivery systems, were synthesized through the microwave-assisted hydrothermal method by varying the reaction parameters (i.e., pressure—10 and 80 bar, reaction time—30 and 60 min) at the temperature of 60 °C. In this manner, four different types of nanocarriers were obtained and used for the subsequent experimental steps.

**Figure 1:**
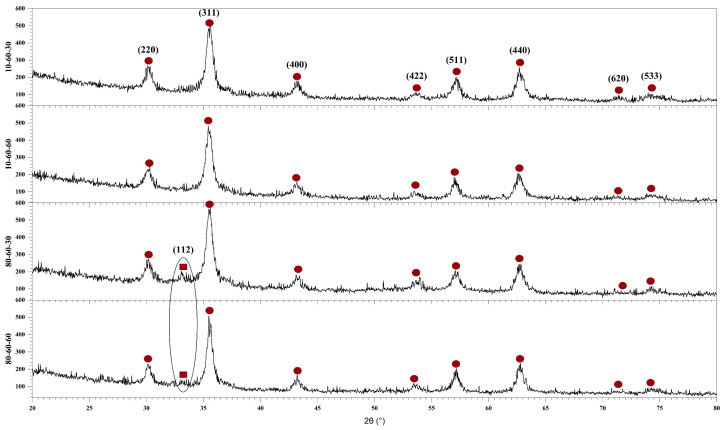
The diffractograms of the four iron oxide nanoparticle samples (10-60-30, 10-60-60, 80-60-30, and 80-60-60) and the Miller indices associated with each diffraction peak (●—Fe_3_O_4_, ■—FeO(OH)).

Therefore, the first step in the characterization flow was to determine the influence of the synthesis parameters on the mineral phase formation, crystallinity, and crystallite size of the obtained nanoparticles. In this context, Figure 1 depicts the diffractograms acquired for the four types of iron oxide nanocarriers used in the development of drug-delivery systems. On one hand, the diffractograms show the formation of a single crystalline phase for the samples 10-60-30 and 10-60-60 (synthesized at lower-pressure conditions), which is demonstrated through the diffraction peaks specific for magnetite (Fe_3_O_4_) in the Fd-3 m cubic crystal system and the associated Miller indices (JCPDS 00-019-0629 [32]). On the other hand, the diffractograms show the formation of two crystalline phases for the 80-60-30 and 80-60-60 samples (synthesized at higher-pressure conditions). Specifically, besides the diffraction peaks characteristic of magnetite, there is one diffraction peak at the 2θ angle of 33° that was attributed to goethite (FeO(OH)) in the Pbnm orthorhombic crystal system (JCPDS 00-029-0713 [33]), which is known to occur at high pressure and temperature conditions [19,25].

Using the acquired X-ray diffractograms, the results were subjected to Rietveld refinement in order to assess the proportion of each crystalline phase, the crystallite size, and the crystallinity of the nanocarriers (Table 2). The results confirm the formation of the goethite phase at higher-pressure conditions, with a much higher proportion formed in the 80-60-30 sample (also shown in the diffractograms through a higher intensity for the corresponding peak). It can also be seen that higher-pressure conditions together with long reaction times lead to a significant increase in the average crystallite size, especially for the goethite phase, due to the crystal growth process. Consequently, longer reaction times lead to a considerable decrease in nanoparticle crystallinity.

**Figure 2:**
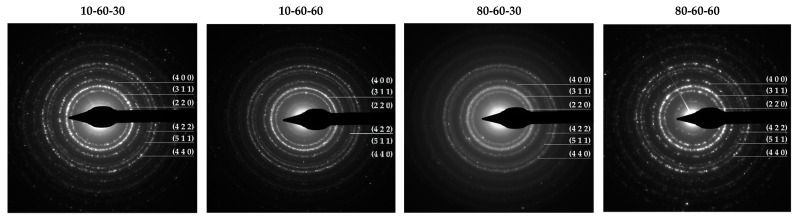
SAED diffraction patterns with the corresponding Miller indices for the iron oxide nanocarriers.

Furthermore, the Miller indices determined by measuring the SAED rings (Figure 2) match the ones from the diffractograms corresponding to the magnetite phase. However, the patterns reveal more diffused diffraction rings for the 80-60-30 sample, which are generally associated with nanoparticles with decreased crystallinity. Thus, considering the results from both XRD and SAED, it could be stated that the presence of the goethite phase increases the overall crystallinity of the samples.

**Figure 3:**
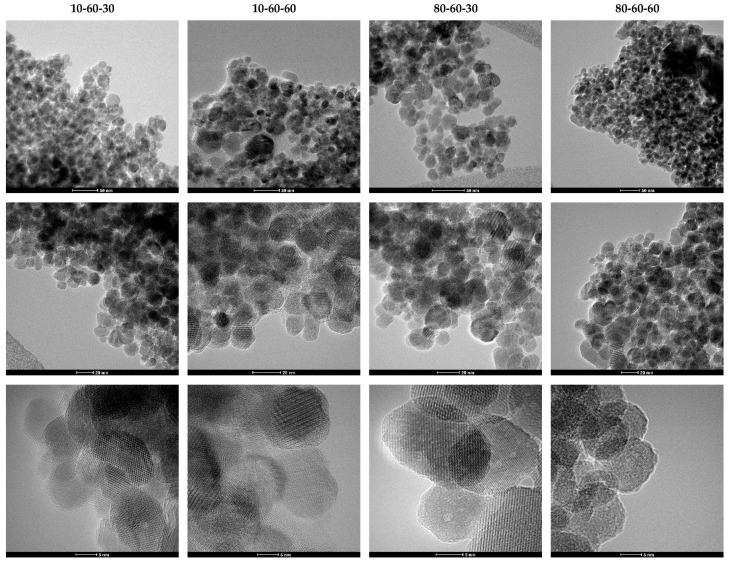
TEM and HR-TEM images at different scale bars for the iron oxide nanocarriers.

**Figure 4:**
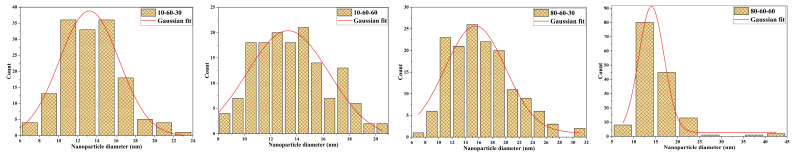
The size distributions of the iron oxide nanocarriers.

TEM analysis was further used for assessing the shape and size of the iron oxide nanocarriers. As can be seen from the TEM and HR-TEM images (Figure 3), the nanoparticles exhibit a quasispherical shape. Additionally, the HR-TEM images reveal a monocrystalline nature of the nanoparticles. The images were subsequently used for estimating the size of the nanocarriers, by measuring 150 nanoparticles in the ImageJ software. The so-obtained results were applied for creating the size distributions which were further fitted in the Origin software using the available Gaussian curve fit (Figure 4). Considering the center (xc value) of the Gaussian fits, the average nanoparticle size is as follows: 10-60-30—13.20 nm, 10-60-60—13.32 nm, 80-60-30—15.45 nm, and 80-60-60—14.03 nm. While there are no significant differences between samples obtained at different reaction times, it can be seen that high-pressure conditions represent the key factor to determine the formation of nanoparticles with broad size distributions.

**Figure 5:**
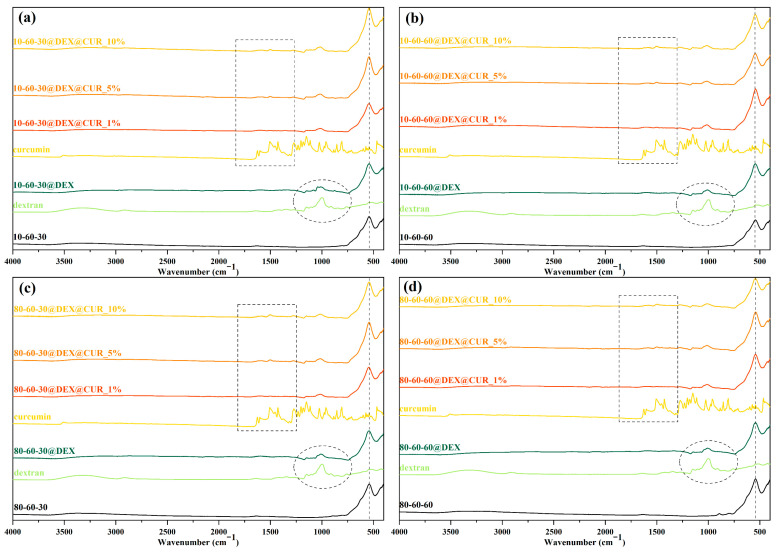
FT-IR spectra for (**a**) 10-60-30 and the associated samples, (**b**) 10-60-60 and the associated samples; (**c**) 80-60-30 and the associated samples; (**d**) 80-60-60 and the associated samples.

Furthermore, FT-IR spectroscopy was utilized for confirming the dextran coating of the iron oxide nanoparticles and the subsequent curcumin loading at different concentrations. In this regard, Figure 5 depicts the FT-IR spectra registered for all 20 samples (pristine, dextran-coated, and curcumin-loaded iron oxide nanoparticles), as well as for dextran and curcumin for comparison. The absorption band at 541 cm^−1^ is characteristic of the Fe-O bond, thus confirming the formation of the iron oxide nanoparticles. The marked circle represents the wavenumber area characteristic for the dextran coating, while the marked rectangle is associated with the curcumin loading, where the intensity of the absorption bands is proportional to the curcumin concentration. Table 3 summarizes the wavenumber for each of the identified absorption bands and the associated bonds. Thus, it can be seen that the identified bonds correspond to the bonds present within the molecular structures of dextran and curcumin.

**Figure 6:**
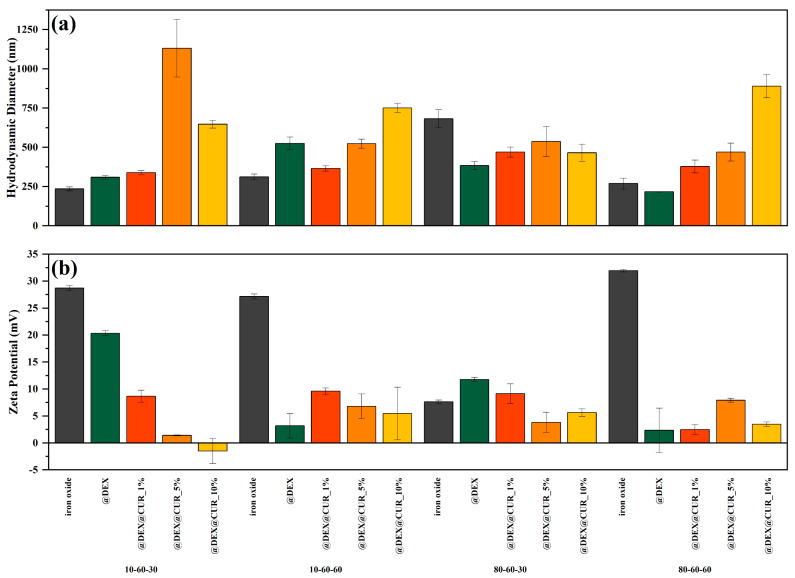
The average hydrodynamic diameter (**a**) and the zeta potential (**b**) values, grouped by the type of the iron oxide nanocarrier.

The DLS and zeta potential measurements were performed for all 20 samples (pristine, dextran-coated, and curcumin-loaded iron oxide nanoparticles) (Figure 6). It can be observed that the general tendency involves the increase in the hydrodynamic diameter and the decrease in the zeta potential values with the addition of dextran followed by curcumin at increasing concentrations. Thus, it can be assumed that the polymer coating and the biosubstance loading lead to a significant reduction in the charge present on the surface of the drug-delivery systems and consequently the interactions with the solvent (water in this case).

**Figure 7:**
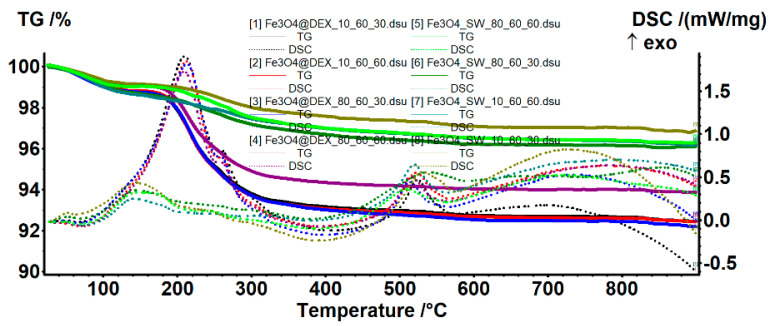
TG-DSC curves for the pristine and the dextran-coated iron oxide nanoparticles.

The TG-DSC analysis was used for estimating the efficiency of dextran coating (Figure 7, Table 4). Both pristine and dextran-coated iron oxide nanoparticles present three mass-loss steps. In the temperature interval of 25–135 °C, all samples present a mass loss of ~1%, accompanied by an endothermic peak with a minimum of ~70 °C. This process is attributed to the water molecules weakly bonded to the surface of the nanoparticles, which are the first to be eliminated. Subsequently, the temperature interval of 135–400 °C leads to the oxidation of magnetite to maghemite (Fe^2+^ to Fe^3+^), together with the degradation of the organic molecules and the elimination of strongly bonded –OH moieties from the nanoparticle surface [34,35]. The predominant effect is exothermic, which appears as a broad, combined effect, with the maximum at ~200 °C. The DSC exothermic peak at ~515 °C is attributed to the specific phase transition from maghemite to hematite, which commonly occurs after 500 °C [29,36].

**Figure 8:**
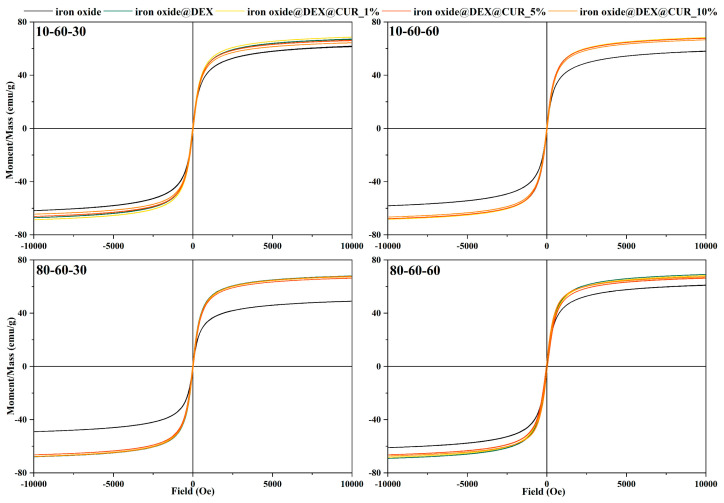
Field-dependent magnetization measurements for the dextran-coated iron oxide nanoparticles loaded with curcumin.

The magnetic properties of the pristine, dextran-coated, and curcumin-loaded iron oxide nanoparticles were determined through the VSM analysis (Figure 8). The results demonstrate the superparamagnetic behavior of the nanoparticles through the S-shaped hysteresis curve with a zero width, as resulted from applying a magnetic field from 10 to −10 kOe. While the saturation magnetization values for the pristine iron oxide nanoparticles range between 50 to 60 emu/g, the addition of the dextran coating seems to increase the saturation magnetization to ~70 emu/g. Furthermore, the curcumin loading does not modify the magnetization of the nanoparticles at any concentrations, thus proving the potential of these drug-delivery systems to be used for the external magnetic field-controlled release of bioactive substances.

**Figure 9:**
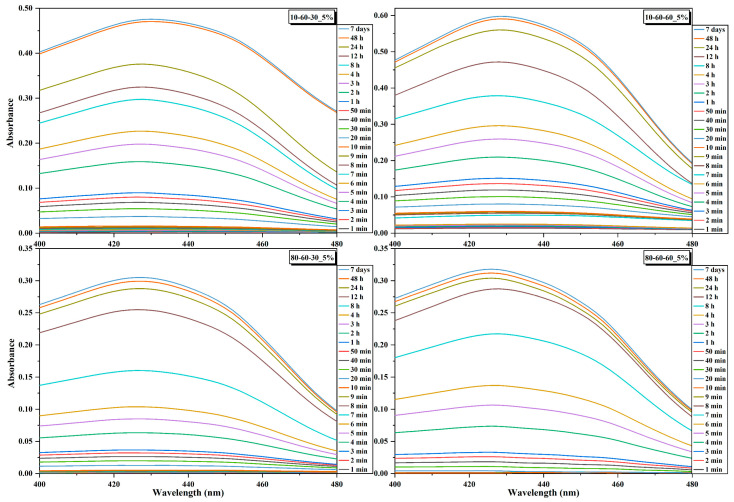
Absorbance signals acquired at selected time points for the dextran-coated iron oxide nanocarriers loaded with 5% curcumin.

**Figure 10:**
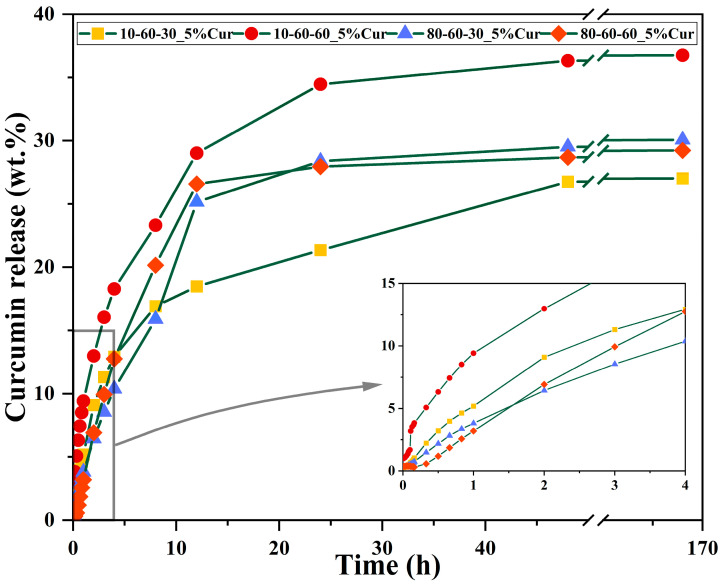
Release profiles with selected timepoints for the dextran-coated iron oxide nanocarriers loaded with 5% curcumin.

The curcumin loading efficiency and the release studies were assessed through UV-Vis spectrophotometry. It can be observed that there are no major differences between the nanocarriers, as the loading efficiency is similar for most of the systems loaded with the same amount of curcumin (Table 5). However, a considerable decrease in the loading efficiency can be observed for the samples 80-60-30 and 80-60-60 with 10% curcumin, which could be explained by a lower surface area that is saturated at 5% curcumin. Therefore, the 5% curcumin samples were further selected for investigating the release behavior (Figure 9 and Figure 10). The 4 h short-term study shows that samples obtained at high-pressure conditions are characterized by a considerably slower release of curcumin, by half, as compared to the lower-pressure counterparts. Moreover, the samples subjected to longer reaction treatments exhibit a slightly faster release, which could also be associated with the increased dimensions of the nanocarriers. The 72 days long-term study shows similar percentages of ~30% of released curcumin for all samples. However, considering that the loading efficiency for the samples obtained at lower-pressure conditions is slightly higher, it is safe to conclude that the amount of released curcumin for these samples is higher (Figure 10). Furthermore, it can be seen that the samples reach a sustained release at the 24 h/48 h time intervals.

**Figure 11:**
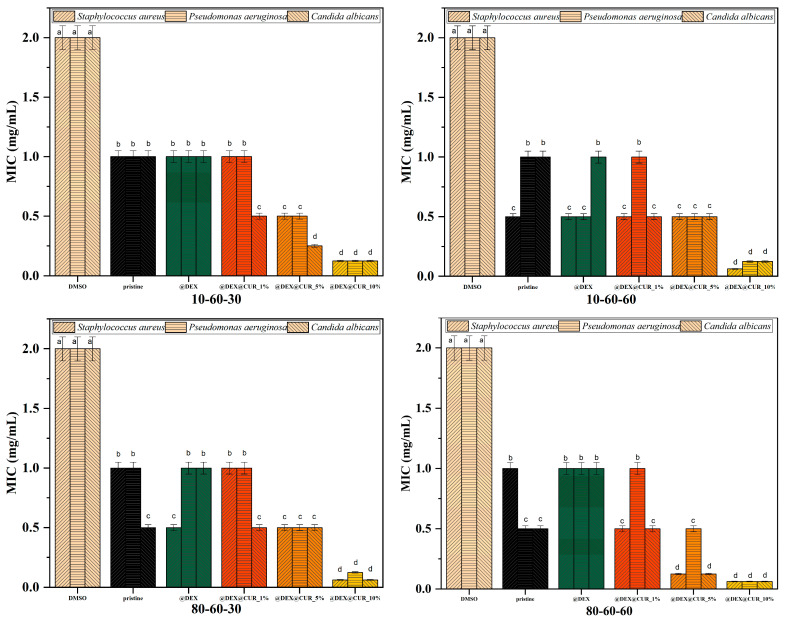
The MIC values registered for each pristine iron oxide and dextran-coated iron oxide nanocarriers loaded with curcumin (different letters indicate significant differences between each sample).

**Figure 12:**
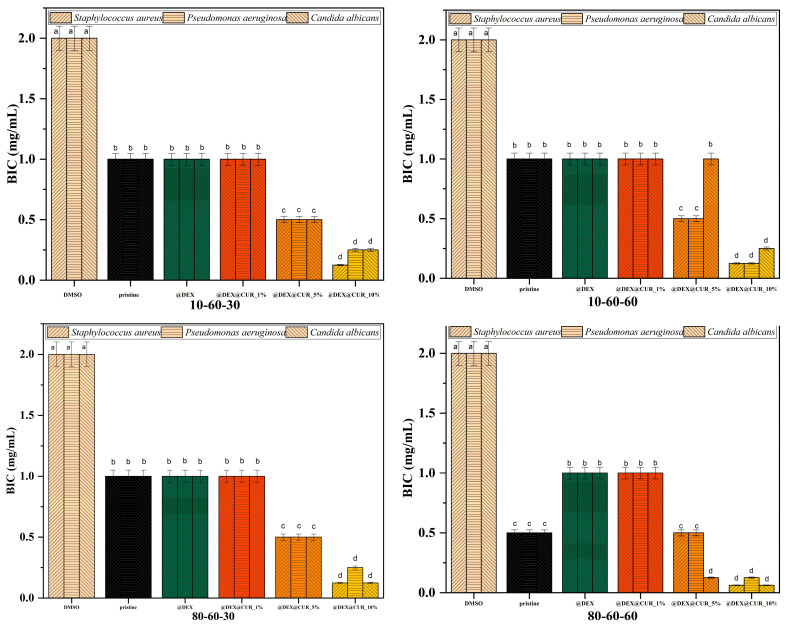
The BIC values registered for each pristine iron oxide and dextran-coated iron oxide nanocarriers loaded with curcumin (different letters indicate significant differences between each sample).

The antimicrobial efficiency of the curcumin-loaded drug-delivery systems is illustrated in Table 6 and Figure 11 and Figure 12, through the inhibition zone, MIC, and BIC values. The overall results demonstrate the capacity of the nanosystems to inhibit the growth of both Gram-positive (*Staphylococcus aureus*) and Gram-negative (*Pseudomonas aeruginosa*) bacteria, as well as the yeast *Candida albicans*.

Results show that the pristine iron oxide nanoparticles have a similar capacity for inhibiting the growth of the microbial strains. However, the dextran coatings do not enhance the antimicrobial efficiency of the iron oxide nanocarriers, in most cases ensuring a microbial proliferative environment. The presence of curcumin within the nanosystems further increases their antimicrobial activity, especially in the case of 10% curcumin loading (Table 6). There are no significant differences between the microbial strains, thus demonstrating the potential of these drug-delivery systems to be used for preventing a wide variety of microbial species.

The MIC evaluation shows similar results as the inhibition zone, with no statistically significant differences between the pristine and dextran-coated iron oxide nanoparticles and enhanced antimicrobial activity for the curcumin-loaded samples. For all types of nanocarriers, it is easily seen that the 10% curcumin-loaded systems present MIC values statistically lower compared to the negative control (Figure 11). Thus, it can be stated that in the case of planktonic growth, the antimicrobial effects are mostly due to the presence of the antimicrobial substance, and not a synergistic effect with the nanoparticles. Furthermore, these results support the idea that the designed dextran-coated iron oxide nanosystems could be an efficient carrier for natural antimicrobial agents, such as curcumin.

We further evaluated the antimicrobial efficiency of the designed nanosystems in biofilm cultures, since it is widely accepted that bacteria in biofilms are more resistant to antimicrobial agents as compared to planktonic cultures. Antibiofilm results were consistent with the data obtained in planktonic cultures. Specifically, the potential of biofilm inhibition was highest for the 10% curcumin-loaded samples (up to four folds), with no statistically significant difference between the pristine, dextran-coated, and 1% curcumin-loaded samples in most cases (Figure 12).

**Figure 13:**
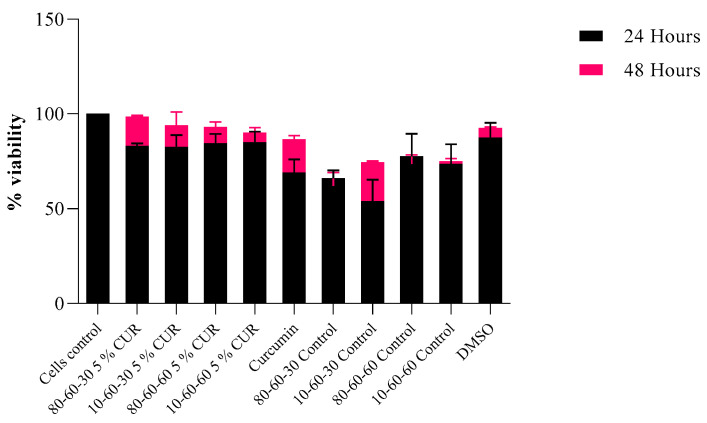
Viability rates of HT-29 cells after 24 h and 48 h of exposure to dextran-coated iron oxide nanocarriers as controls and dextran-coated iron oxide nanocarriers loaded with 5% curcumin (*p* = 0.03).

Citotoxicity tests revealed that the designed nanocarriers show no or limited interference with eukaryotic-cultured cells. Specifically, the results obtained on HT-29 intestinal cells indicate a high percentage of viability (Figure 13), especially for the 48 h time point of exposure to the tested nanosystems. As for the 24 h mark, viability was over 75% for the curcumin-loaded samples and over 60% for the dextran-coated controls. This could be explained by the initial shock that cells tend to suffer after a novel compound is introduced into the culture media. However, for the 48 h mark, the viability rates were over 89% for the curcumin-loaded samples and over 70% for the dextran-coated controls, with a significant increase in viability observed, especially for curcumin-loaded samples.

**Figure 14:**
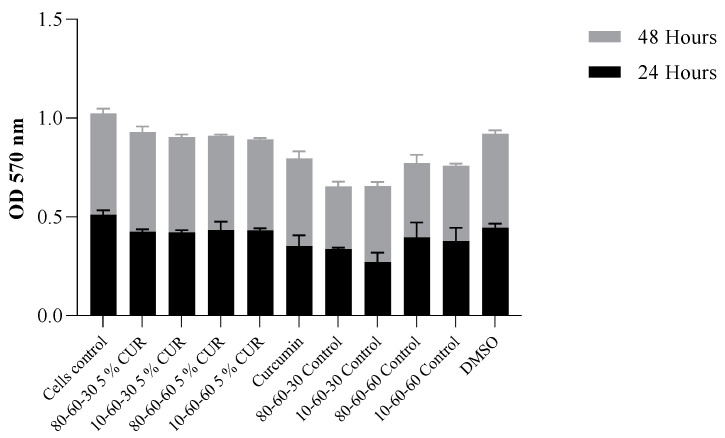
Graphic representation of MTT OD values at 24 h and 48 h of exposure to dextran-coated iron oxide nanocarriers as controls and dextran-coated iron oxide nanocarriers loaded with 5% curcumin (*p* = 0.034).

Viability results obtained for the controls indicate a high level of positive impact in biocompatibility provided by the dextran coating of the nanoparticles (over 15% improvement of cellular survival rate). The ODs obtained by the MTT assay are in correlation, indicating a normal metabolic cell function, with minimal cytotoxicity present at 48 h of incubation with the curcumin-loaded samples (Figure 14).

## 4. Discussion

The present study aimed to develop efficient and biocompatible nanostructured drug-delivery systems with potential applicability within antimicrobial therapies. The experimental design consisted of three main steps, namely the microwave-assisted hydrothermal synthesis of iron oxide nanocarriers with varying synthesis parameters; the coating of the nanocarriers with a widely used biocompatible polymer, i.e., dextran; and the loading of curcumin, a natural bioactive substance with well-known anticarcinogenic, antioxidant, and antimicrobial properties, at three different concentrations.

The microwave-assisted hydrothermal method is a widely used technique for the synthesis of numerous nanoparticles, such as hydroxyapatite [37,38,39], zinc oxide [40,41,42], and copper oxide [43,44], as it allows for the control of the nanoparticle morphology, size, and porosity. While recent years have witnessed an increased interest in applying this method in developing iron oxide nanoparticles [29,45,46,47], its utilization is still limited, with most studies focusing on more readily available methods (i.e., coprecipitation). As it was indicated by the XRD and SAED results, the microwave-assisted hydrothermal method ensured the synthesis of highly crystalline iron oxide nanoparticles consisting of one or two mineral phases. Furthermore, the TEM and DLS analyses demonstrated the possibility of modulating their size and size distribution by varying the synthesis parameters, which could further aid the control of the release profiles. Generally, the application of nanoparticles with larger sizes is often regarded as a safer approach, as their agglomeration and accumulation are prevented [48]. Additionally, larger nanoparticles are also able to avoid rapid body clearance [49]. However, iron oxide nanoparticles are synthesized with sizes lower than ~10 nm, as increasing the nanoparticle size is a relatively difficult process [50]. In this regard, our results proved the possibility of obtaining iron oxide nanoparticles with larger sizes, thus proving the capacity of the microwave-assisted hydrothermal method to provide a facile and efficient means of overcoming the previously mentioned issues.

Furthermore, dextran was added as a coating for the iron oxide nanocarriers to enhance their stability and biocompatibility, prevent nanoparticle agglomeration, and limit cytotoxicity. Dextran is a highly biocompatible and nontoxic polysaccharide with antioxidant, anti-inflammatory, and antithrombotic properties that is widely used in numerous pharmaceutical and biomedical applications [51]. Thus, its potential is demonstrated through the great number of studies available in the literature that apply dextran as a coating for iron oxide nanoparticles for drug-delivery and diagnostics applications [52,53,54,55,56]. In contrast to other reports [57], another important aspect proven through this study by the VSM analysis is the capacity of the dextran coating to enhance the magnetic properties of the iron oxide nanocarriers. This effect could be explained through the reduction in the surface disorder generally responsible for significant changes in the magnetic properties of nanoparticles by the organic coating, which bonds with the surface cations and aligns the nanoparticle spins [58]. Moreover, the saturation values of the present nanostructures are similar to those obtained for superparamagnetic iron oxide nanoparticles, namely close to 60 emu/g [59]. Thus, their suitability for further hyperthermia-based drug-release applications was demonstrated.

Finally, the biosubstance chosen for this study was curcumin, the biphenolic bioactive compound of the *Curcuma longa* plant [60]. In the biomedical area, curcumin has been widely used as an antioxidant, anti-inflammatory, anticancer, antiapoptotic, and neuroprotective agent [61,62,63]. In the present study, curcumin was investigated for its antimicrobial properties against Gram-positive, Gram-negative, and fungal strains [64,65]. While previously published papers have demonstrated a considerably greater sensitivity for Gram-positive than Gram-negative bacteria [66], our results indicated similar antimicrobial efficiency towards all the tested strains, thus proving the potential of the current drug-delivery systems to be used for a wide spectrum of microbial species. 

From another point of view, the available literature demonstrated the sustained release of curcumin for long periods of over 15 days [67], which is mainly caused by its increased hydrophobicity that prevents drug release [68,69]. In this manner, curcumin-loaded nanomaterials should be designed for applications that require long-term release, such as implant coatings, which could represent a future direction of the present study. Additionally, release studies should also investigate the curcumin release from our dextran-coated iron oxide nanocarriers in different pH media, which could help direct the area of the organism for which they are most suitable.

## 5. Conclusions

The present study demonstrated the potential of the microwave-assisted hydrothermal method for developing iron oxide nanostructures with varying and controllable properties, especially in terms of mineral phase composition and nanoparticle crystallinity, size, and size distribution. Subsequently, the dextran coating considerably enhanced the stability, magnetic behavior, and biocompatibility of the inorganic nanoparticles. The loading with curcumin showed the potential of the drug-delivery systems in preventing microbial infections and biofilm formation. Thus, besides their direct administration, these nanosystems could also be applied as coatings for various implantable devices, such as bone implants, catheters, meshes, and wound dressings, which could benefit a broader segment of patients with infection risks. In this context, future research directions should focus on the next stage of nanostructured antimicrobial-agent applicability.

## Figures and Tables

**Table 1:** The microwave-assisted hydrothermal treatment parameters used for the synthesis of the iron oxide nanoparticle supports.

Sample Code	Pressure [bar]	Temperature [°C]	Treatment Time [min]	Stirring [%]
10-60-30	10	60	30	10
10-60-60	10	60	60	
80-60-30	80	60	30	
80-60-60	80	60	60	

**Table 2:** The proportions of the crystalline phases, the average crystallite size, and the crystallinity of the iron oxide nanocarriers.

Sample	Crystalline Phase Proportions [%]	Average Crystallite Size ± SD [nm]	Crystallinity [%]
10-60-30	magnetite–100	9.42 ± 0.22	20.56
10-60-60	magnetite–100	9.04 ± 0.23	18.55
80-60-30	magnetite–66.60 goethite–33.40	9.04 ± 0.68 2.09 ± 0.19	27.99
80-60-60	magnetite–94.10 goethite–5.90	11.16 ± 0.42 415.93 ± 1115.23	18.23

**Table 3:** The absorption bands identified within the FT-IR spectra and the associated bonds.

Type of Bond	Wavenumber (cm^−1^)
Fe-O	541
C-O stretching (primary alcohol)	1078, 1146
C-O stretching (alkyl aryl ether)	1276
C=C stretching (alkene)	1583
O-H (phenol)	1427
C-H bending	1495

**Table 4:** The mass losses registered and the associated thermal effects according to the TG-DSC curves.

Sample	Mass Loss (%)			Dextran-Loaded (%)	Thermal Effects (°C)		
	25–135 °C	135–400 °C	Residual		Endothermic	Exothermic—Maghemite	Exothermic—Hematite
**10_60_30**	0.85	1.58	96.84	-	66.5	143.6	532.4
**10_60_30@DEX**	1.20	5.66	92.46	4.52	67.2	209.9	524.5
**10_60_60**	1.34	1.70	96.16	-	71.4	142.8	520.1
**10_60_60@DEX**	1.19	5.77	92.41	3.90	70.3	212.2	521.8
**80_60_30**	1.31	2.06	96.10	-	70.2	146.0	538.7
**80_60_30@DEX**	1.22	5.78	92.17	4.09	68.1	213.1	512.8
**80_60_60**	1.01	2.03	96.28	-	69.9	147.9	508.9
**80_60_60@DEX**	0.99	4.70	93.87	2.50	70.1	209.4	515.0

**Table 5:** The amount of added curcumin and the loading efficiency for the dextran-coated iron oxide nanocarriers.

Sample		Amount of Loaded Curcumin (mg)	Loading Efficiency (%)	Curcumin Released after 7 Days (%)
**10-60-30**	1%	5.5	55	-
	5%	12.5	25	27.02
	10%	60.0	60	-
**10-60-60**	1%	4.0	40	-
	5%	11.5	23	36.77
	10%	30.0	30	-
**80-60-30**	1%	6.0	60	-
	5%	7.5	15	30.08
	10%	15.0	15	-
**80-60-60**	1%	3.5	35	-
	5%	8.0	16	29.24
	10%	15.0	15	-

**Table 6:** The inhibition zone for each pristine iron oxide and dextran-coated iron oxide nanocarriers loaded with curcumin.

Sample	Inhibition Zone (mm)		
	*Staphylococcus aureus*	*Pseudomonas aeruginosa*	*Candida albicans*
DMSO	1	1	1
10-60-30	6	5	6
10-60-30@DEX	3	5	5
10-60-30@DEX@CUR_1%	6	7	6
10-60-30@DEX@CUR_5%	7	7	7
10-60-30@DEX@CUR_10%	7	7	7
10-60-60	6	5	6
10-60-60@DEX	6	6	6
10-60-60@DEX@CUR_1%	5	5	7
10-60-60@DEX@CUR_5%	6	6	5
10-60-60@DEX@CUR_10%	7	7	8
80-60-30	6	6	6
80-60-30@DEX	6	5	6
80-60-30@DEX@CUR_1%	5	7	6
80-60-30@DEX@CUR_5%	6	6	7
80-60-30@DEX@CUR_10%	8	7	8
80-60-60	7	5	6
80-60-60@DEX	6	6	6
80-60-60@DEX@CUR_1%	8	7	7
80-60-60@DEX@CUR_5%	7	6	6
80-60-60@DEX@CUR_10%	8	7	8

## Data Availability

Not applicable.
